# Sema4C mediates EMT inducing chemotherapeutic resistance of miR-31-3p in cervical cancer cells

**DOI:** 10.1038/s41598-019-54177-z

**Published:** 2019-11-27

**Authors:** Li Jing, Wang Bo, Feng Yourong, Wang Tian, Wang Shixuan, Wu Mingfu

**Affiliations:** 1 Department Gynecology, Cancer Biology Research Center, Tongji Hospital, Tongji Medical College, Huazhong University of Science and Technology, Wuhan, Hubei 430030 P.R. China; 2 Wuhan women and children’s center, Wuhan, Hubei 430030 P.R. China

**Keywords:** Cervical cancer, Cancer therapeutic resistance

## Abstract

Sema4C, the target of many miRNAs, is involved in EMT-mediated chemotherapeutic resistance of many malignant tumors. However, the underlying upstream regulatory mechanisms of Sema4C-induced EMT and Sema4C-mediated drug resistance are still unclear. The aim of this study was to explore the potential role of miR-31-3p/Sema4C in regulating EMT in cisplatin-resistant (CR) cervical cancer cells. High expression levels of Sema4C were more frequently found in cervical cancer tissues and were associated with poor prognosis, whereas miR-31-3p was significantly downregulated in cervical cancer tissues, which was associated with shorter disease-free and overall survival. Overexpression of miR-31-3p inhibited malignant behaviors and EMT of cervical cancer cells *in vitro*. Furthermore, miR-31-3p was identified to directly target Sema4C, and upregulation of miR-31-3p reversed EMT-mediated biological functions, including cisplatin resistance of Sema4C in cervical cancer cells. These results suggest that Sema4C promoted EMT-mediated cisplatin resistance in cervical cancer cells and that this effect was inhibited by overexpression of miR-31-3p. Thus, silencing Sema4C or overexpression of miR-31-3p could be a novel approach to treat drug resistance to chemotherapy in cervical cancers.

## Introduction

Cervical cancer (CC) is a common malignancy of the female reproductive tract and the leading cause of cancer-related deaths in women worldwide^1^. There were approximately 527,000 new cases of cervical cancer worldwide in 2012, of which approximately 266,000 died. Due to the improvement of cervical cancer prevention and screening systems, the incidence of cervical cancer is higher in developing countries than the 7.8/100000 in developed countries such as the United States. Because most diagnosed patients are already at an advanced stage, the mortality of cervical cancer is high^2^. Patients with advanced/recurrent cervical cancer have a very poor prognosis, with a 1-year survival rate of only 10–20%^3^. Chemotherapy is one of the standard treatments for cervical cancer, which can obviously inhibit tumor growth and improve prognosis^4^. Cisplatin (CDD), a small molecule platinum compound, has been used to treat cervical cancer^5^ since as early as the late 20th century, and so far still promises to be the most effective drug for treating advanced/recurrent cervical cancer^6^. However, resistance to cisplatin, which is acquired intrinsically or during cancer progression, may seriously compromise the efficacy of CDD and lead to chemotherapy failure and poor prognosis^7^. Therefore, it is of great theoretical and clinical significance to investigate the potential molecular mechanism of drug resistance to chemotherapy for cervical cancer.

Epithelial to mesenchymal transition (EMT) refers to the complex biological processes involved in the transformation of epithelial cells into cells with mesenchymal features. Emerging bodies of evidence have indicated that EMT is closely associated with chemotherapy resistance through the involvement of EMT-associated transcription factors in human cancers including human breast cancer, cervical cancer, epithelial ovarian cancer, and hepatocellular carcinoma^8–13^. The transcription factor and EMT inducer Twist1 is involved in ovarian cancer metastasis and chemo-resistance^9^. Paclitaxel-resistant (PR) epithelial ovarian cancer A2780 cells presented an interstitial phenotype by upregulating phosphoinositide 3-kinase (PI3K)^10^, and gemcitabine-resistant hepatocellular carcinoma cells (HCC) were shown to have EMT characteristics^11^. In breast cancer cells, downregulation of Foxc2 as a key determinant of interstitial and stem cell characteristics inhibits interstitial phenotype, invasion, and metastasis and reduces chemotherapy resistance^12^. In cervical cancer cells, downregulation of astrocyte-elevated gene-1 (AEG-1) reverses EMT and increases chemotherapy drug sensitivity^13^.

Sema4C, originally called M-SemaF, was identified as a brain-rich class 4 transmembrane vertebrate semaphorin by its homology to the Sema domain^14^. In our previous studies, tumor-associated lymphatic endothelial cells (LECs) were found for the first time to produce soluble Sema4C (sSema4C) through MMP cleavage, and increased serum sSema4C was detected in patients with breast cancer and cervical cancer and in those with metastasis. It was finally found that sSema4C promoted lymphatic metastasis by plexin B2-MET signaling-mediated EMT of tumor cells^15^. Zhou *et al*. found that in renal HK2 cells, Sema4C induces EMT by inhibiting E-cadherin expression and upregulating Vimentin. In renal tubular epithelial cells, downregulation of Sema4C reverses TGF-β1-induced EMT by inhibiting the phosphorylation of P38 MAPK, whereas overexpression of Sema4C induces EMT by promoting the phosphorylation of P38 MAPK^16^. Increasing studies have indicated that Sema4C plays important regulatory roles in tumor invasion, metastasis and EMT and that Sema4C the target of a number of microRNAs (miRNAs) including miR-125b, miR-138, miR-31, miR-25-3p, and miR-205 is involved in EMT-mediated chemotherapeutic resistance of many malignant tumors, including breast cancer, lung cancer, cervical cancer, and HCC^17–20^. However, the underlying upstream regulatory mechanisms of Sema4C-induced EMT and Sema4C-mediated drug resistance are still unclear.

In this study, high expression levels of Sema4C were more frequently found in cervical cancer tissues and were associated with poor prognosis, whereas miR-31-3p was significantly downregulated in cervical cancer tissues. MiR-31-3p was identified to directly target Sema4C and mediated the biological functions including drug resistance of Sema4C in cervical cancer cells. Furthermore, miR-31-3p overexpression inhibited Sema4C-induced EMT to influence tumor cell migration and increase chemo-sensitivity to CDD. Therefore, it is of great theoretical and innovative significance to clarify the functional role and regulatory mechanism of Sema4C in cervical cancer cells.

## Results

### Sema4C is upregulated in cervical cancer tissues and cancer cell lines

**Figure 1:**
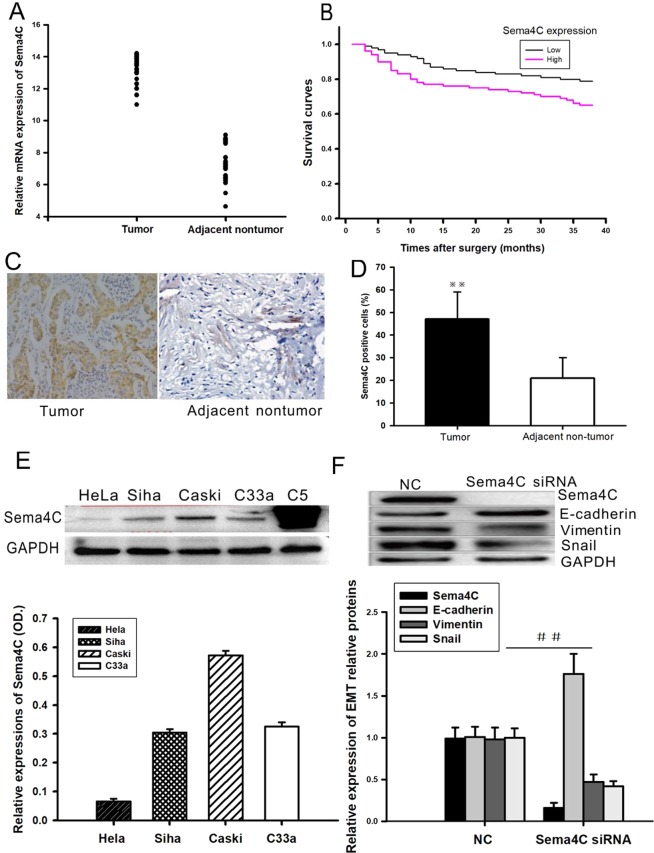
Down-regulation of Sema4C expression reverses EMT in cervical cancer cells. (**A**,**C**,**D**) Immune-histochemical method and RT-PCR were used to detect the up-regulation of Sema4C in cervical cancer tissues. Data are presented as mean ± SD. *P* = 0.0141, n = 52. (**B**) Patients with high Sema4C expression levels had shorter OS and DFS than those with low expression levels of Sema4C. Data are presented as mean ± SD. P values for overall survival (OS) and disease-free survival (DFS) were evaluated with Kaplan-Meier survival curves and were compared by the log-rank test. **P = 0.008, n = 26. (**E**) Western blot showed that the up-regulation of Sema4C in four CC cell lines Hela, Siha, Caski and C33a, C5 as the positive control. Data are presented as mean ± SD. n = 3. (**F**) Western blot showed that down-regulation of Sema4C in Caski cells of cervical cancer resulted in increased expression of epithelial markers E-cadhein, while decreased expression of interstitial markers Vimentin and EMT-related transcription factor Snail. (**G**) Graphic representation of Sema4C expression in the four CC cell lines. (**H**) Graphic representation of relative expression of EMT proteins in the Sema4C treated Caski cells. Data are presented as mean ± SD. ^##^*P* = 0.022, n = 3.

The mRNA levels of Sema4C were determined by qRT-PCR in a previous cohort of 52 paired CC clinical specimens. Compared with that in adjacent and healthy cervical tissues, Sema4C mRNA was significantly upregulated in 36 (69.23%) CC tissues (Fig. 1A and S1). Additionally, Sema4C protein expression in CC tissues detected by immunohistochemical staining analysis was dramatically increased when compared with that in adjacent noncancerous tissues (Fig. 1C,D). Clinicopathologic analysis showed that Sema4C mRNA expression was strongly associated with para-cervical invasion, FIGO staging, and histopathological classification (Table 1). We performed a Kaplan-Meier survival analysis to evaluate the prognostic value of Sema4C in patients with CC. As shown in Fig. 1B, patients with high Sema4C expression levels had shorter OS and DFS than those with low expression levels of Sema4C. The 1-year and 3-year OS rates of patients with high Sema4C expression were 77 and 65%, respectively, which were significantly lower than those of patients with low Sema4C expression (89 and 79%, respectively) (Fig. 1B). These results suggest that Sema4C is upregulated in CC and is associated with poor prognosis of patients with CC.

**Table 1:** Correlation between Sema4C mRNA expression and miR-31-3p expression and clinicopathologic features of patients with cervical cancer.

Factors	N	Sema4C expression		P	miR-31-3p expression			P
		Low (n = 26)	High (n = 26)		Low (n = 26)	High (n = 26)		
**Age (yr)**								
<40	22	12	10	0.779	9		13	0.400
>40	30	14	16		17		13	
**Tumor size**								
<4 cm	31	15	16	1	18		13	0.573
>4 cm	21	11	10		9		12	
**FIGO stage**								
I/II	36	22	14	0.033	15		21	0.035
III/IV	16	4	12		11		5	
**Lymphatic metastasis**								
Yes	11	2	9	0.038	1		8	0.032
No	41	24	17		25		18	
**Paracervical invasion**								
>2/3	18	5	13	0.039	11		6	0.036
<2/3	34	21	13		15		20	
**Histological classification**								
G1	14	5	9	0.029	10		4	0.031
G2/G3	38	21	17		22		16	

Underlined values signify *P* < 0.05.

Consistent with Sema4C expression in CC tissues, Sema4C expression was also detected in four different CC cell lines, HeLa, Siha, Caski and C33a (Fig. 1E). Importantly, among the four CC cell lines, the expression level of Sema4C was the highest in the Caski cell line and the lowest in the HeLa cell line. These results indicated that abnormal expression of Sema4C was correlated with the occurrence and development of cervical cancer, and the Caski cell line was selected as the cell model for further investigation into the functional mechanism of Sema4C.

Previous studies have demonstrated that Sema4C is highly expressed in breast cancer and lung cancer cells and can prompt the invasion and metastasis of tumors by inducing EMT^21,22^. We performed experiments to assess whether Sema4C enhanced EMT in CC cells. Western blot results revealed that downregulation of Sema4C in cervical cancer Caski cells remarkably increased the protein levels of the epithelial marker E-cadherin, whereas the protein levels of the mesenchymal marker vimentin and the EMT-related transcription factor Snail were significantly reduced (Fig. 1F). The results above suggest that Sema4C plays an important regulatory role in EMT in cervical cancer cells.

### Sema4C is directly targeted by miR-31-3p in cervical cancer cells

To reveal the underlying miRNA regulatory mechanisms by which a specific miRNA exerts its functional effects on CC cells and Sema4C expression, we predicted and identified the potential targeting miRNAs of the Sema4C gene by searching three databases, namely, TargetScan, miRDB, and miRanda. Bioinformatics analysis showed that six miRNAs Let-7g, miR-181b, miR-125a, miR-31-3p, miR-19a and miR-34a might directly target Sema4C.

**Figure 2:**
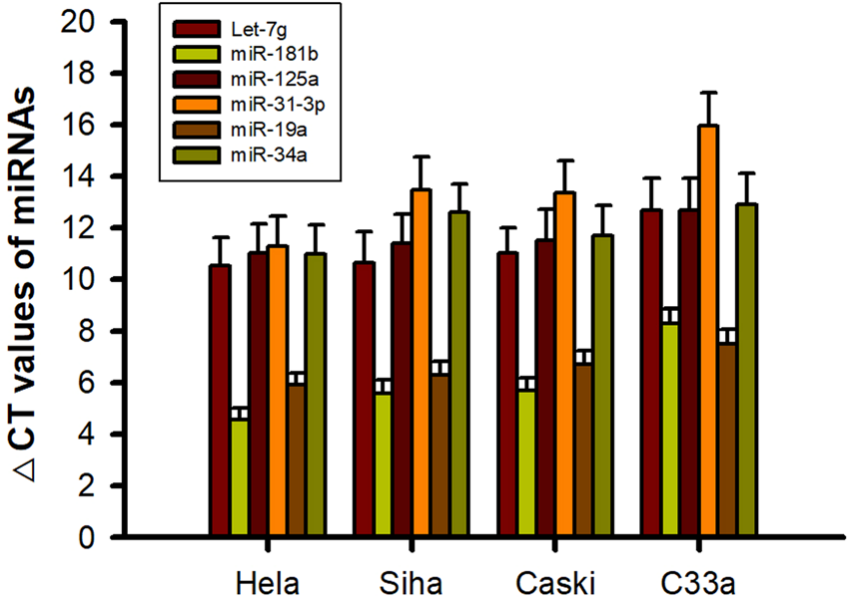
The expression of candidate target miRNAs for Sema4C in the cervical cancer cells. Bioinformatics analysis showed that the six miRNAs including Let-7g, miR-181b, miR-125a, miR-31-3p, miR-19a and miR-34a might be a candidate target of Sema4C.

Consistent with the high expression of Sema4C in cervical cancer cells and the highest expression in Caski cells, the candidate target miRNAs were detected to have low or no expression in cervical cancer cells and the lowest expression was in Caski cells. Real-time RT-PCR results showed that in the four cervical cancer cell lines, miR-181b expression increased, while expression of the other five miRNAs decreased, but downregulation of miR-31-3p expression was the most remarkable and the lowest in Caski cells. Thus, miR-31-3p was selected as the candidate target miRNA of Sema4C (Fig. 2).

**Figure 3:**
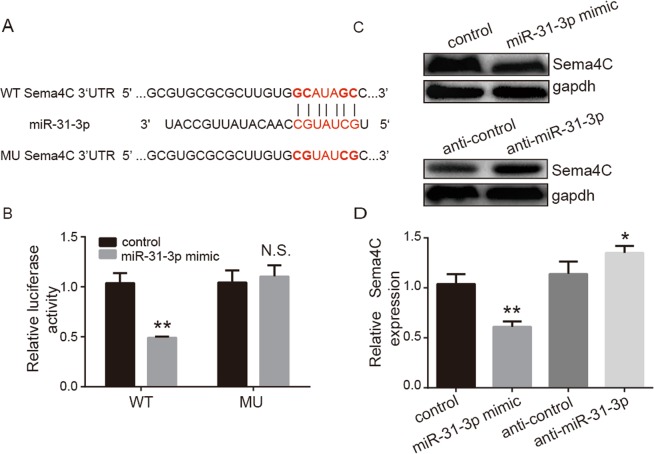
Target relationship between Sema4C and miR-31-3p. (**A**) Bioinformatics target gene prediction revealed the complementary binding sites between Sema4C and miR-31-3p. (**B**) Double luciferase reporter system experiments showed that miR-31-3p mimic could significantly reduce the relative fluorescence intensity of wild type Sema4C-3′UTR, but had no effect on the relative fluorescence intensity of mutant Sema4C-3′UTR. Data are presented as mean ± SD. Paired student’s t test was analyzed. **P = 0.011, n = 3. (**C**,**D**) Western blot assays showed that miR-31-3p mimic in cervical cancer Caski cells could significantly down-regulate Sema4C expression at protein level. MiR-31-3p inhibitor could up-regulate Sema4C expression. Data are presented as mean ± SD. P value was analyzed by simple one-way ANOVA analysis, *P = 0.005 and **P = 0.008, n = 3.

To further functionally verify that Sema4C was directly targeted by miR-31-3p in CC, we investigated whether miR-31-3p directly interacted with the predicted 3′-UTR of Sema4C by a dual-luciferase reporter assay. A complementary sequence of miR-31-3p was found in the 3′-UTR of Sema4C (Fig. 3A). The results showed that miR-31-3p overexpression or mimics significantly inhibited the luciferase activity of wt-Sema4C-3′-UTR compared with the NC miRNA (Fig. 3B). In contrast, the luciferase activity of mu-Sema4C-3′-UTR was not affected by miR-31-3p or the NC miRNA, indicating that Sema4C was directly targeted by miR-31-3p. Consistent with the above findings, western blotting also demonstrated that overexpression of miR-31-3p markedly suppressed the protein levels of Sema4C (Fig. 3C,D). The above functional studies showed that overexpression of miR-31-3p could downregulate Sema4C expression at the protein level.

### MiR-31-3p is downregulated in cervical cancer tissues and correlated with a poor prognosis

**Figure 4:**
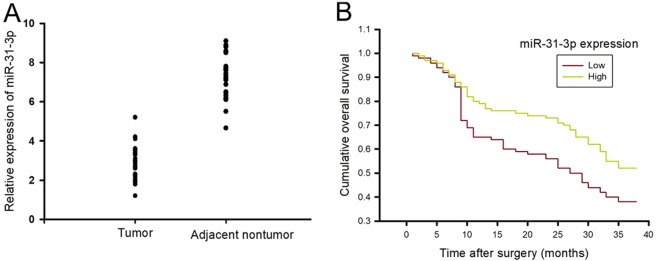
MiR-31-3p is down-regulated in cervical cancer tissues and is correlated with a poor prognosis. (**A**) RT-PCR was used to detect the downregulation of miR-31-3p in cervical cancer tissues, Data are presented as mean ± SD. n = 52; (**B**) Patients with low miR-31-3p expression levels had shorter OS and DFS than those with high expression levels of miR-31-3p. Data are presented as mean ± SD. P value for OS was evaluated with Kaplan-Meier survival curves and was compared by the log-rank test, *P* = 0.001, n = 26.

To investigate the expression and significance of miR-31-3p in cervical cancer tissues, the expression level of miR-31-3p in 52 matched pairs of CC and adjacent nontumor tissues was first detected. We found that miR-31-3p expression was significantly downregulated in 73.08% (38 of 52) of CC tissues when compared with corresponding adjacent nontumor tissues (Fig. 4A and S2).

To further confirm the relationship between miR-31-3p expression and clinicopathologic features, 52 patients with CC were divided into 2 subgroups based on the median level of miR-31-3p in CC tissues. Interestingly, clinicopathologic analysis showed that miR-31-3p expression was strongly correlated with paracervical invasion, FIGO staging, and histopathological classification (Table 1). In addition, Kaplan-Meier survival analysis showed that the patients with CC who had low miR-31-3p expression levels had significantly shorter OS and DFS than those with high miR-31-3p expression levels. The 1-year and 3-year OS rates of patients with low miR-31-3p expression were 65 and 38%, respectively, which were significantly lower than those in patients with high miR-31-3p expression (78 and 52%, respectively; *P* = 0.001) (Fig. 4B).

### Overexpression of miR-31-3p reverses EMT in cervical cancer Caski cells

**Figure 5:**
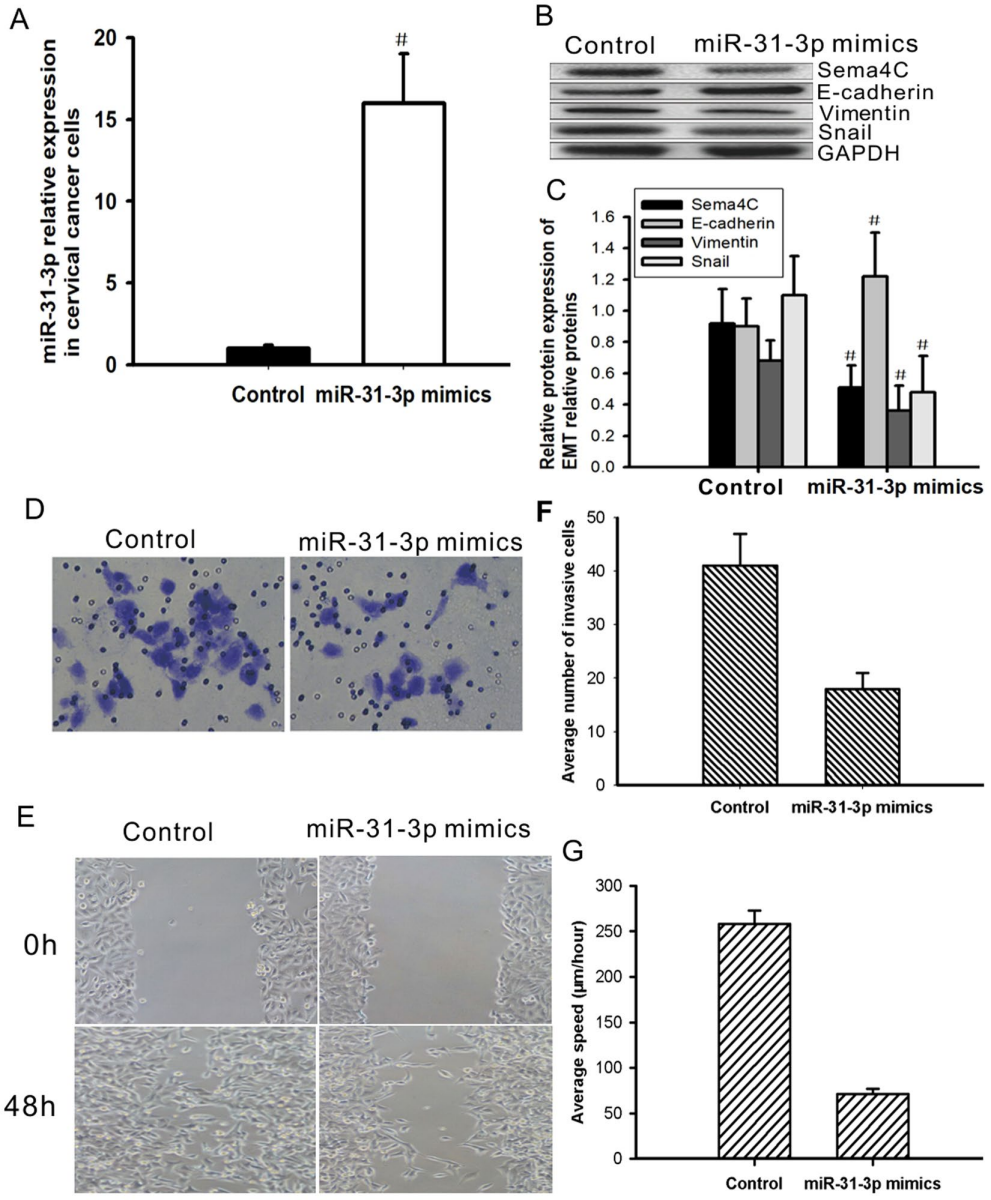
Overexpression of miR-31-3p reverses EMT and inhibits cell migration and invasion in cervical cancer cells. Caski cells were transfected with miR-31-3p mimic or negative control mimic. (**A**) After 24 hours of transfection, the expression level of miR-31-3p in Caski cells was detected by real time PCR. Data are presented as mean ± SD, Paired student’s t test was analyzed. ^#^*P* = 0.0061, n = 3. (**B**,**C**) Expression levels of Sema4C, E-cadherin, Vimentin and Snail proteins in Caski cells were detected by western blotting after 48-hour transfection. Data are presented as mean ± SD. P value was analyzed by simple one-way ANOVA analysis, ^#^*P* < 0.05, n = 3. (**D**–**G**) After 24 hours of transfection, cell invasion assay and wound healing assay were used to detect the ability of cell migration and invasion. GAPDH is the internal references. Data are presented as mean ± SD, n = 3.

To study whether miR-31-3p is involved in EMT of cervical cancer cells, a miR-31-3p mimic was transfected into Caski cells to observe whether overexpression of miR-31-3p could affect the expression of EMT markers. The miR-31-3p mimic significantly increased the expression level of miR-31-3p in cervical cancer Caski cells (Fig. 5A) and significantly increased the mRNA and protein levels of E-cadherin, while it downregulated the mRNA and protein levels of Vimentin and Snail (Fig. 5B,C). Furthermore, after transfection with the miR-31-3p mimic, overexpression of miR-31-3p inhibited tumor cell invasion and migration in Caski cells (Fig. 5D–G). These results suggest that overexpression of miR-31-3p can reverse EMT and inhibit tumor cell invasion and migration of cervical cancer cells.

### Sema4C mediates the functional effects of miR-31-3p and EMT in CC cells

**Figure 6:**
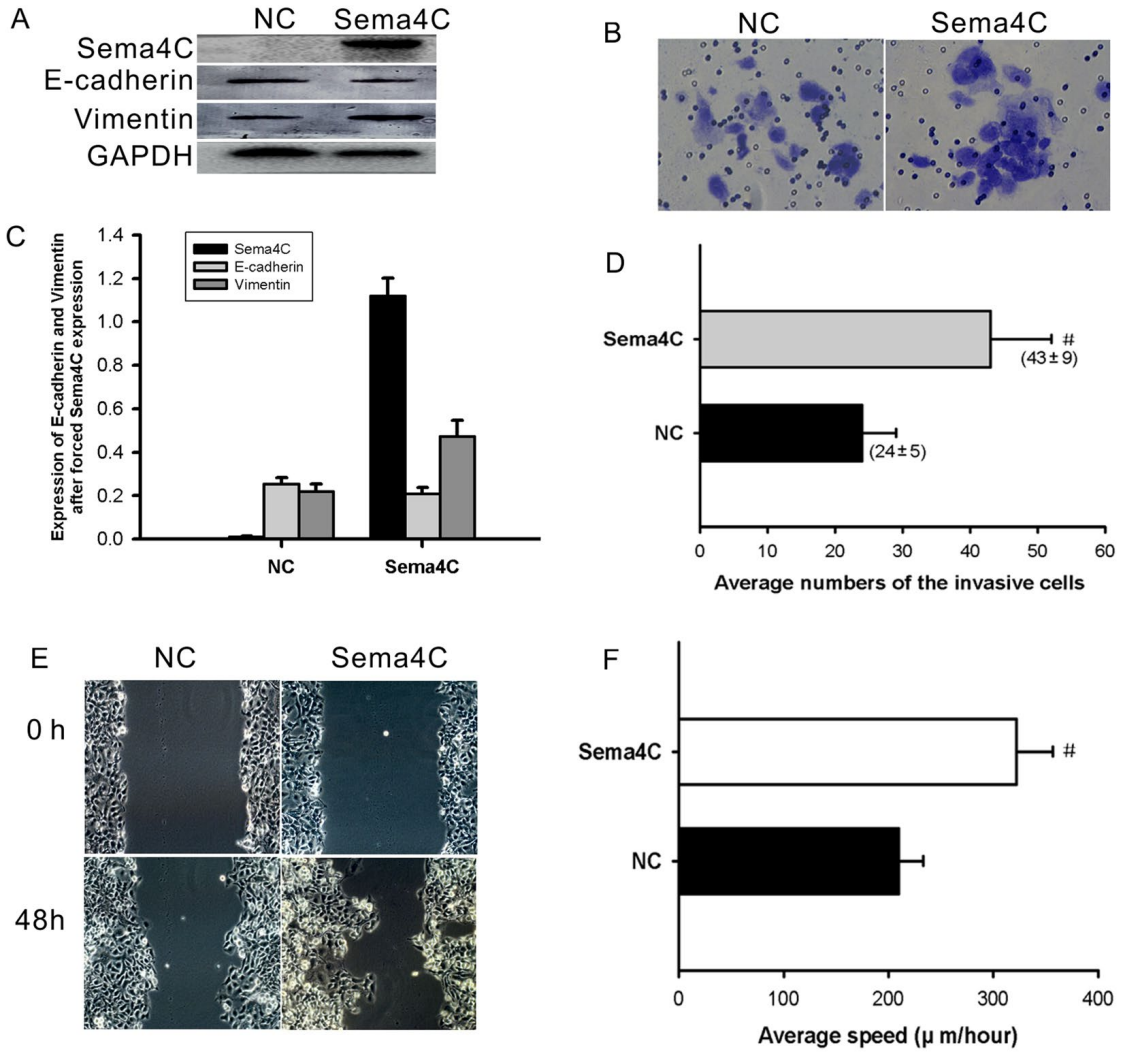
Sema4C mediates the functional effects of miR-31-3p and EMT in CC cells. (**A**,**C**) The repressive effects of miR-31-3p on EMT were rescued by overexpression of Sema4C. (**B**,**D**–**F**) Sema4C restoration abrogated the inhibitory effects on migration and invasion of Caski cells induced by miR-31-3p. Data are presented as mean ± SD. P value was analyzed by paired student’s t test, ^#^*P* = 0.019 for cell migration and ^#^*P* = 0.014 for cell invasion, n = 3.

To ascertain whether miR-31-3p elicits inhibitory effects on cervical cancer cells through Sema4C, Sema4C was restored by overexpression plasmid in Caski cells stably overexpressing miR-31-3p. Subsequent western blotting results confirmed the overexpression of Sema4C in miR-31-3p–overexpressing Caski cells (Fig. 6A,C). As expected, Sema4C restoration abrogated the inhibitory effects of miR-31-3p on the migration and invasion of Caski cells (Fig. 6B,D–F). Likewise, the repressive effects of miR-31-3p on EMT were rescued by overexpression of Sema4C, leading to molecular changes associated with downregulation of E-cadherin and increased expression of vimentin (Fig. 6A,C). Thus, these data provide evidence that re-expression of Sema4C could rescue the miR-31-3p–mediated migration, invasion, and EMT of CC cells.

### Downregulation of Sema4C or overexpression of miR-31-3p increases the sensitivity of cervical cancer cells to cisplatin

**Figure 7:**
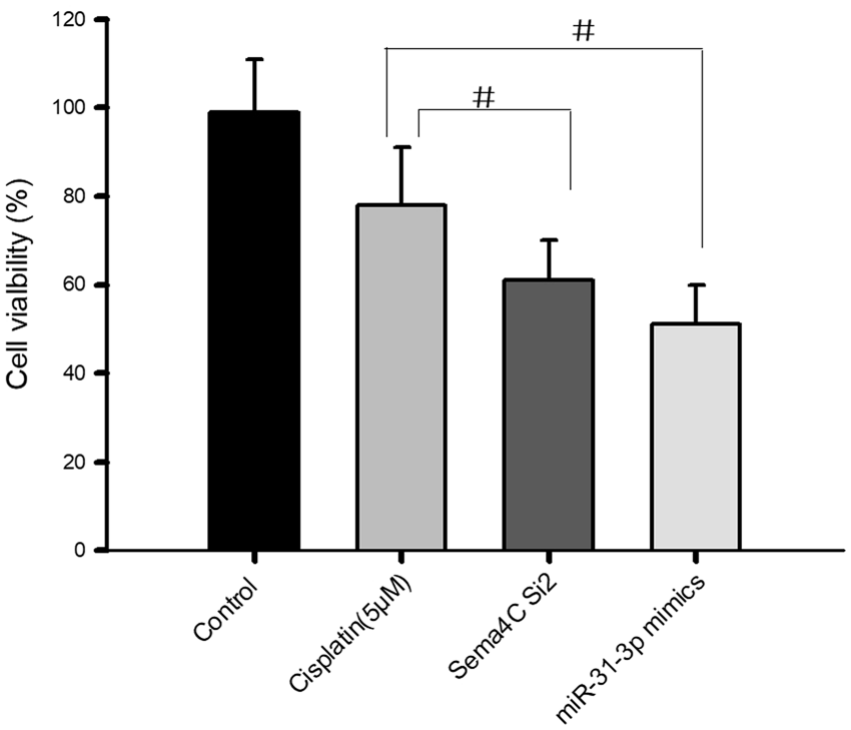
Down-regulation of Sema4C or up-regulation of miR-31-3p increases the sensitivity of cervical cancer cells to cisplatin. After 24 h transfection with Sema4C si2 (25 nM) or miR-31-3p mimic (50 nM), CCK8 assay was performed after 5 μM cisplatin treatment. Data are presented as mean ± SD. P value was analyzed by simple one-way ANOVA analysis, ^#^*P* = 0.021, n = 3.

Tumor cells have not only have enhanced migration and invasion ability but also drug resistance characteristics^23^. A CCK8 assay was used to detect cisplatin resistance in cervical cancer cells after treatment with Sema4C siRNA or miR-31-3p mimic. As shown in Fig. 7, after 24 h of pretreatment with Sema4C si2 (25 nM) or miR-31-3p mimic (50 nM), the inhibitory effect of 5 μM cisplatin on the growth of cervical cancer cells was significantly enhanced compared with cisplatin alone. The drug inhibition rates of cisplatin alone, Sema4C si2 combined with cisplatin and miR-31-3p mimic were 24%, 44% and 51%, respectively. The above results indicated that downregulation of Sema4C or upregulation of miR-31-3p could significantly increase the sensitivity of cervical cancer cells to cisplatin.

## Discussion

Previous studies have demonstrated that Sema4C increases cell proliferation and migration and regulates EMT in tumors^19,20^. Sema4C overexpression inhibits E-cadherin expression and upregulates Vimentin to induce EMT in hepatocellular carcinoma^24^, breast cancer^20^, CR cervical cancer^17^ and PR lung cancer^18^. In the present study, high Sema4C expression levels were more frequently found in CC tissues and CC cell lines, and abnormal Sema4C expression was associated with poor prognosis. In addition, downregulation of Sema4C remarkably reversed EMT in Caski cells.

Increasing evidence indicates that abnormal miRNA expression is found in different tumors and that the aberrant dysfunction of miRNAs plays a crucial role in the occurrence, invasion and metastasis of tumors^21,25^, especially the regulation of chemotherapeutic resistance and chemotherapy-related EMT^22,24,26^. Compared with those in matched healthy liver tissues, in HCC, especially aggressive HCC, notably reduced miR-205 expression levels were associated with poor clinicopathologic features, such as shorter DFS and OS. The stemness of HCC was promoted by the downregulation of miR-205, which targets the PLC β1 gene, which then upregulates CD24 expression^24^. The recovery of Sema4C expression reversed the repressive effects of miR-205 on HCC malignant behaviors such as migration, invasion, and EMT. In non-small-cell lung cancer (NSCLC) cells, miR-138 overexpression significantly suppressed cell growth and reversed EMT by targeting GIT1 and Sema4C, whereas silencing GIT1 and Sema4C by siRNA suppressed tumor cell proliferation and reversed EMT, which indicated that the inhibitory effects of miR-138 were partly rescued by abnormal GIT1 and Sema4C expression^19^. In this study, bioinformatics analysis showed that six miRNAs, namely, Let-7g, miR-181b, miR-125a, miR-31-3p, miR-19a and miR-34a might directly target Sema4C. According to the detected expression patterns of Sema4C and the miRNAs in CC cells, miR-31-3p was selected as the candidate targeting miRNA of Sema4C. Furthermore, further verification and functional studies showed that Sema4C was directly targeted by miR-31-3p in CC and that overexpression of miR-31-3p could downregulate Sema4C expression at the protein level. These data indicate that understanding the novel roles and molecular mechanism of miR-205, miR-138, miR-31-3p and Sema4C in tumor progression has great theoretical and innovative significance, which may provide new targets or prognostic biomarkers for the treatment of cancer, including CC.

Sema4C, the target of many miRNAs including miR-125b, miR-25-3p, miR-205, miR-138, and miR-31, is involved in the EMT-mediated chemotherapeutic resistance of many malignant tumors, including breast cancer, cervical cancer, HCC, and lung cancer^17–20^. Ectopic expression of miR-125b in breast cancers and lung cancers^18,20^ and of miR-25-3p in CC^17^ notably reversed the EMT phenotype and regulated PR- and CR-induced EMT by downregulating Sema4C expression. PR cells were sensitized by enforced expression of miR-125b or Sema4C depletion, while miR-25-3p overexpression sensitized CR cells to cisplatin in HeLa CR cells and suppressed tumor growth in mice^17^. Stable overexpression of miR-125b in stable paclitaxel-resistant (PR) breast cancer MCF-7 PR and SKBR3 PR cells and lung cancer A549 PR cells suppressed the growth of tumor xenografts in immunodeficient mice^18,20^. These results suggest that specific miRNAs including miR-31-3p are important regulators of tumor EMT and chemo-resistance and that upregulating miRNAs or targeting Sema4C could serve as a new approach to reverse tumor chemotherapy resistance.

Increasing evidence has shown that miR-31 is upregulated in many cancerous tissues, including colorectal, lung and cervical cancers^23,27–30^. The upregulation of miR-31 in colorectal tumor tissue and cancer cell lines accelerates the proliferation, motility and invasiveness of tumor cells, whereas silencing miR-31 results in tumor cell apoptosis^23,27^. In lung adenocarcinoma, miR-31 expression is increased both in adenocarcinoma cells compared to human lung epithelial cell lines and in NSCLC cells compared to normal lung tissue^28^. Moreover, miR-31 is correlated with lung cancer progression. The expression of miR-31 in stage I lung adenocarcinoma was 5.7-fold higher than that in normal lung tissues and increased to 13.5-fold in stage II and III and 35-fold in stage IV, suggesting that miR-31 was related to the occurrence of lung adenocarcinoma^28^. In CC, miR-31-3p could be used as a molecular marker to predict the sensitivity of patients with locally advanced CC to radiotherapy and chemotherapy^29^. In RAS and KRAS wild-type metastatic colorectal cancer patients, miR-31-3p was used as a molecular marker to predict the response of anti-EGFR therapy^30,31^. Therefore, miR-31-3p is currently limited to descriptive analysis of sequencing results, and the functional role for miR-31-3p has not been reported. In this study, we found that decreased miR-31-3p expression in CC tissues was associated with shorter DFS and OS. The EMT phenotype was reversed, and CR cells were sensitized to cisplatin by miR-31-3p overexpression or by targeting Sema4C in Caski cells. Furthermore, compared with the control group, enforced miR-31-3p expression notably inhibited the invasion and migration of tumor cells, suppressed Sema4C and Snail expression, and increased E-cadherin expression in Caski-CR cells. These results suggested that miR-31-3p served as an important regulator of EMT and chemo-resistance in CC. Thus, upregulating miR-31-3p expression could be a new method to treat CDD-resistant CC.

In summary, we discovered that Sema4C is more frequently upregulated, while miR-31-3p expression was obviously decreased in CC tissues and cancer cell lines. Sema4C and miR-31-3p are distinctly correlated with shorter DFS and OS. Ectopic expression of Sema4C restored the suppressive effect of overexpressed miR-31-3p. Our results indicate that direct targeting of Sema4C by miR-31-3p promoted the metastatic behaviors and induced EMT of CC cells. This study offers new insights into the mechanism of Sema4C involving CC progression and suggests that miR-31-3p serves as a promising therapeutic target and novel prognostic indicator for patients with CC by targeting Sema4C. Overexpression of miR-31-3p or downregulation of Sema4C may be a new approach to reverse the resistance to chemotherapy in cervical cancer.

## Methods

### Patients and specimens

The collection of patients and specimens was similar with the method described previously^32,33^. Fifty-two paired human cervical cancer (CC) and adjacent nontumor cervical tissues were collected from the Tongji Hospital Affiliated with the Tongji Medical College of Huazhong University of Science and Technology (HUST) between January 2018 and December 2018. Histopathologic diagnosis was performed according to the revised 2018 FIGO cervical cancer staging [International Gynecologic Cancer Society (IGCS), Kyoto, Japan]. The tissues were stored at −80 °C or embedded in paraffin. No patients received any preoperative chemotherapy or radiotherapy. Informed consent was obtained from each patient, and the Ethics Committee of Tongji Hospital approved all protocols of this study.

### Cell culture and miRNA transfection

Human cervical cancer cell lines (HeLa, Caski, Siha, and C33a) were purchased from the Institute of Biochemistry and Cell Biology (Chinese Academy of Sciences, Shanghai, China). Siha and C33a cells were cultured in complete DMEM (Gibco, Thermo Fisher Scientific, Waltham, MA, USA) containing 10% fetal bovine serum (Thermo Fisher Scientific) at 37 °C in a humidified incubator with 5% CO_2_. HeLa and Caski cells were cultured in RPMI-1640 (Gibco, Thermo Fisher Scientific, Waltham, MA, USA) supplemented with 10% heat-inactivated fetal bovine serum (HyClone, Logan, UT, USA). Caski cells were cultured in increasing concentrations of cisplatin (Sigma, St. Louis, MO) for more than 6 months to establish cisplatin-resistant (CR) cell lines Caski CR cells. Cells were transiently transfected with the above vectors using Polo Deliverer 3000 Transfection Reagent (Shanghai R&S Biotechnology Co.) in accordance with the manufacturer’s protocol.

### MiRNA target prediction and dual-luciferase reporter assay

The analysis of miR-31-3p predicted targets was performed with TargetScan, miRDB, and miRanda algorithms. The 3′-UTR sequence of Sema4C, which was predicted to interact with miR-31-3p and a corresponding mutated sequence of Sema4C that contained the miR-31-3p binding sites were synthesized and cloned into the psiCHECK-2 dual-luciferase reporter vector (Gene Copoeia) called wild-type (wt)-Sema4C 3′-UTR (wt) and mutant (mt)-Sema4C 3′-UTR (mutated).

Subsequently, luciferase reporter assay was performed described previously^33^. The cells were cultured in 24-well plates and cotransfected with the wt or mt Sema4C 3′-UTR vector together with 50 nM miR-31-3p mimics or NC miRNA with Lipofectamine 2000 Reagent (Thermo Fisher Scientific). Forty-eight hours after cotransfection, firefly and Renilla luciferase activities were measured with the Dual-Luciferase Reporter Assay Kit (Promega, Madison, WI, USA) according to the manufacturer’s protocol. The ratio of firefly to Renilla luciferase was used to normalize the luciferase activity.

### Wound-healing assay

This method was performed as described previously^17^. Cells (1 × 10^6^ cells per well) were plated on 6-well plates. Cells were cultured in medium containing 1% bovine serum albumin (BSA). A sterile 200 μl pipette tip was used to create a gap by scraping the cells. The migration process was monitored (after identification of each wounded zone) in six areas immediately and 48 h after wounds were made using an inverted microscope (Nikon TMS-F, 301655, Nikon, Tokyo, Japan) installed with a digital camera (Nikon Digital shot DS-L1, Nikon). Cell migration data were expressed as the ratio of the change in gap width divided by the initial gap width.

### Cell invasion assays

The protocol was performed as described previously^17^. Briefly, 1 × 10^4^ cells in 100 μL serum-free medium were seeded in a Transwell apparatus (Costar, Corning, NY, USA) containing a fibronectin-coated polycarbonate membrane insert. Medium (500 μL) containing 10% fetal bovine serum was added to the lower chamber as a chemoattractant. After an 8-h incubation at 37 °C in a 5% CO_2_ incubator, the insert was washed extensively with PBS, and the top surface of the insert was wiped clean with a cotton swab to remove the cells. Cells on the lower surface were fixed with methanol and stained with crystal violet. Cells adhering to the lower surface were counted using five predetermined fields. All assays were independently repeated in triplicate.

### RNA isolation, reverse transcription and qRT-PCR

This method was performed as described previously^17,32^. Total RNA was extracted from fresh-frozen cervical cancer tissues or cell lines with TRIzol reagent (Gibco, Thermo Fisher Scientific, Waltham, MA, USA). cDNA synthesis was performed with 2 mg total RNA using a reverse transcription kit (Takara Bio, Kusatsu, Japan). Real-time, quantitative RT-PCR (qRT-PCR) was performed with an All-in-One miRNA qRT-PCR Detection Kit (GeneCopoeia) on an ABI 7700 Real-Time PCR System (Thermo Fisher Scientific). Real-time qRT-PCR was performed with SYBR Green Master Mix. All miRNA RT-primers for hsa-Let-7g, hsa-miR-181b, hsa-miR-125a, hsa-miR-31-3p, hsa-miR-19a, hsa-miR-34a, and small nuclear RNA U6 were purchased from RiboBio Co., Ltd. U6 was used as an internal control for microRNA expression levels, and GAPDH was used for mRNA expression levels. The ΔCt and ΔΔCt methods were used to analyze gene expression relative to endogenous control expression.

### Western blot analysis

This method was performed as described previously^17^. The cells grown on plates were trypsinized, and detached cells were collected by centrifugation. The cell pellet was lysed with cold lysis buffer supplemented with protease inhibitors. Protein samples (30 µg) from each cell lysate were equivalently loaded on a precast gel and used for electrophoresis. Gels were subsequently blotted onto nitrocellulose membranes (0.45 μM; Bio-Rad, Hercules, CA), followed by blocking nonspecific binding with a solution containing 1 × PBS, 0.1% Tween-20, and 5% nonfat dry milk powder at room temperature for 1 h. Membranes were incubated with the primary antibodies anti-E-cadherin, anti-Snail, anti-Vimentin, anti-GAPDH (Cell Signaling Technology, Danvers, MA, USA) and anti-Sema4C (Abcam, Cambridge, MA, USA) at 4 °C overnight. After extensive washing with TBST, horseradish peroxidase (HRP)-conjugated secondary antibodies (Bio-Rad) were incubated for 1 h at room temperature. Bands were detected with an enhanced chemiluminescence kit (Super Signal West Pico Substrate; Pierce, Rockford, IL, USA). Quantification of signal intensities was performed by densitometry on a Xerox scanner using NIH ImageJ software (ImageJ, Bethesda, MD).

### Transfection

This method was performed as described previously^17^. Briefly, to evaluate the effect of Sema4C knockdown, cells seeded in 6-well plates were subjected to transfection with 25 nM Sema4C siRNA (si2) or control siRNA using Lipofectamine 2000. The sequences used for Sema4C siRNA are as follows: Sema4C siRNA (si2), 5′-UAU AGG UGG CUC CAU CAA GUC CUG U-3′ (RiboBio Co., Ltd., Guangzhou, China). To evaluate the effect of miR-31-3p overexpression, cells were transfected with 50 nM miR-31-3p mimic or the nonspecific control (RiboBio Co., Ltd., Guangzhou, China) using lipofectamine RNAiMAX reagent (Invitrogen) following the manufacturer’s protocol. The cells seeded in 24-well plates were used for transfection with 50 nM miR-31-3p inhibitor (for knockdown of miR-31-3p) or the nonspecific control using DharmaFect Transfection Reagent (Dharmacon, Lafayette, CO) following the manufacturer’s protocol. The cells were subjected to analysis by luciferase reporter assay. For 3′-UTR luciferase reporter assays, a fragment of Sema4C 3′-UTR was amplified by PCR and cloned into the psiCHECK-2 vector (named wt). The miR-31-3p binding site within the Sema4C 3′-UTR was mutated by site-directed mutagenesis using the GeneTailor Site-Directed Mutagenesis System (Invitrogen, Guangzhou, China), yielding the mutant Sema4C 3′-UTR (mt). The wt or mt vector or the control psiCHECK-2 vector, were co-transfected with miR-31-3p mimics or inhibitors into cells in 48-well plates, and then the cells were collected for luciferase assay 48 h after transfection. The Dual-Luciferase Reporter Assay System (Promega) was used for the analysis of luciferase activity, which was normalized to the firefly luciferase activity.

### Establishment of stable miR-31-3p overexpressing cells

This method was performed as described previously^17^. Briefly, lentiviral vectors for miR-31-3p overexpression were purchased from GeneChem (Shanghai, China). A lentiviral vector that expresses a noncoding RNA was used as the control. Caski CR cells were seeded in each well of 24-well plates (5 × 10^4^ cells in each well) and infected with miR-31-3p (Lv miR-31-3p) or control lentiviral vector (control LEV) at a multiplicity of infection of 10 (10 infectious units for each target cell). After 72 h of infection, the cells were selected with puromycin, and miRNA levels were quantified using qRT-PCR.

### Immunohistochemistry

The protocol was performed as described previously^17^. Briefly, tumor sections (5 μm thick) were processed using standard deparaffinization and rehydration protocols. Following rehydration, antigen retrieval was performed by submerging the slides in 10 mmol/L sodium citrate buffer (pH 6.0) and heated using a pressure cooker maintained at 95 °C for 20 min followed by a 20-min cooling. The sections were then washed with PBS and blocked with 1% BSA/2% goat serum before incubation with anti-Sema4C antibody (Cell Signaling Technology). Subsequently, the sections were incubated with biotinylated secondary antibody followed by HRP-conjugated streptavidin. The 2,4-diaminobenzidine (DAB) substrate was then applied to the slides, followed by counterstaining with hematoxylin.

### Cell Counting Kit-8 (CCK-8) assay

This method was performed for drug sensitivity as described previously^33^. Briefly, 5 × 10^4^/ml Caski cells in the logarithmic growth stage were inoculated to 96-well plates (100 μL/well) and cultured in the incubator for 24 h. On the second day, cisplatin (5 μM), Sema4C si2 (25 nM) and miR-31-5p mimics (50 nM) were added into the cells. The drug toxicity was tested after 48 h. Subsequent to being incubated at 37 °C in a 5% CO_2_ incubator for 2 h, 10 µl CCK-8 reagent was added into each well and incubated at 37 °C for additional 4 h. The optical density (OD) at 450 nm for each well was determined using an ELISA reader (Bio-Rad Laboratories, Inc., Hercules, CA, USA). Drug inhibition rate (%) = [1- (mean of OD dosing group − mean of OD blank group/mean of OD solvent control group − mean of OD blank group)] × 100%. All experiments were repeated in triplicate.

### Statistical analysis

All Data are presented as mean ± SD. Statistical analyses were conducted using SPSS software (v.21.0; IBM, Armonk, NY, USA). Treatment groups were analyzed by either Student’s t-test or simple one-way ANOVA using Prism version 5 (GraphPad Software, Inc., San Diego, CA, USA). The Fisher’s exact test was used to analyze the relationship between Sema4C mRNA expression and miR-31-3p expression and clinic-pathologic characteristics. Overall survival (OS) and disease-free survival (DFS) were evaluated with Kaplan-Meier survival curves and were compared by the log-rank test. Differences with P < 0.05 were considered statistically significant.

### Ethical approval and informed consent

#### Ethical approval

The experiment is not involved in the animal experiments, so not applicable. However, approval from the Institutional Ethical Board (IRB) in the Tongji Hospital affiliated to Tongji Medical College of Huazhong University of Science and Technology was obtained in this study.

#### Accordance

The methods were carried out in accordance with the relevant guidelines and regulations.

#### Informed consent

Prior written consent of each patient for the use of clinical materials for research purposes was signed.

## Supplementary information

Supplementary information
