# Supplementary material for: Sema4C mediates EMT inducing chemotherapeutic resistance of miR-31-3p in cervical cancer cells
Source: Sci Rep. 2019 Nov 27;9:17727. doi: 10.1038/s41598-019-54177-z (PMC6881343; doi:10.1038/s41598-019-54177-z)
Supplement: Supplementary file 1 — Supplementary information [file 41598_2019_54177_MOESM1_ESM.docx]

**Sema4C mediates EMT inducing chemotherapeutic resistance of miR-31-3p in cervical cancer cells**

Li Jing ^1#^, Wang Bo ^1#^, Feng Yourong ^1^, Wang Tian, Shixuan Wang ^1^*, and Wu Mingfu ^1^*

^1^Cancer Biology Research Center, Tongji Hospital; Tongji Medical College, Huazhong University of Science and Technology, Wuhan, Hubei 430030, P. R.China

Supplementary file 1

Table 1: CT Value of Sema4C expression in 36 (69.23%) CC tissues by RT-PCR in the figure 1A.

| cases | Tumor | adjacent non-tumor |  |  |  |
| --- | --- | --- | --- | --- | --- |
| 1 | 14 | 8.9 |  |  |  |
| 2 | 12 | 9.1 |  |  |  |
| 3 | 11 | 8.86 |  |  |  |
| 4 | 13 | 8.82 |  |  |  |
| 5 | 14.1 | 8.56 |  |  |  |
| 6 | 14.2 | 8.67 |  |  |  |
| 7 | 13.1 | 8.77 |  |  |  |
| 8 | 13.7 | 8.81 |  |  |  |
| 9 | 13.6 | 8.69 |  |  |  |
| 10 | 13.5 | 8.56 |  |  |  |
| 11 | 13.9 | 6.56 |  |  |  |
| 12 | 13.8 | 5.46 |  |  |  |
| 13 | 13.45 | 6.23 |  |  |  |
| 14 | 12 | 6.12 |  |  |  |
| 15 | 14.11 | 6.35 |  |  |  |
| 16 | 13.88 | 6.243 |  |  |  |
| 17 | 11.6 | 6.98 |  |  |  |
| 18 | 13.23 | 7.1 |  |  |  |
| 19 | 12.6 | 7.23 |  |  |  |
| 20 | 12.3 | 7.34 |  |  |  |
| 21 | 12.98 | 7.45 |  |  |  |
| 22 | 13.5 | 7.68 |  |  |  |
| 23 | 13.87 | 6.45 |  |  |  |
| 24 | 13.56 | 7.12 |  |  |  |
| 25 | 11 | 6.1 |  |  |  |
| 26 | 13.99 | 4.62 |  |  |  |
| 27 | 11.26 | 4.78 |  |  |  |
| 28 | 12.11 | 4.59 |  |  |  |
| 29 | 12.34 | 7.39 |  |  |  |
| 30 | 13.42 | 8.01 |  |  |  |
| 31 | 14.45 | 8.54 |  |  |  |
| 32 | 14.34 | 7.79 |  |  |  |
| 33 | 11.98 | 8.67 |  |  |  |
| 34 | 11.87 | 8.21 |  |  |  |
| 35 | 13.56 | 5.34 |  |  |  |
| 36 | 12.28 | 6.16 |  |  |  |
|  |  |  |  |  |  |

Supplementary file 2

Table 2: CT Value of miR-31-3p expression in 73.08% (38 of 52) CC tissues using RT-PCR in the figure 4A.

| cases | Tumor | adjacent notumor |  |  |  |  |
| --- | --- | --- | --- | --- | --- | --- |
| 1 | 2 | 8.9 |  |  |  |  |
| 2 | 2.1 | 8.8 |  |  |  |  |
| 3 | 2.2 | 8.86 |  |  |  |  |
| 4 | 3 | 8.81 |  |  |  |  |
| 5 | 1.2 | 8.5 |  |  |  |  |
| 6 | 3.6 | 8.6 |  |  |  |  |
| 7 | 4.2 | 9.1 |  |  |  |  |
| 8 | 2.7 | 7.8 |  |  |  |  |
| 9 | 2.8 | 7.6 |  |  |  |  |
| 10 | 3.3 | 7.7 |  |  |  |  |
| 11 | 3.4 | 6.5 |  |  |  |  |
| 12 | 4.1 | 5.5 |  |  |  |  |
| 13 | 5.2 | 6.23 |  |  |  |  |
| 14 | 2.6 | 6.11 |  |  |  |  |
| 15 | 2.9 | 6.33 |  |  |  |  |
| 16 | 2.3 | 6.23 |  |  |  |  |
| 17 | 2.7 | 6.88 |  |  |  |  |
| 18 | 3.3 | 7.11 |  |  |  |  |
| 19 | 3.5 | 7.22 |  |  |  |  |
| 20 | 2.8 | 7.32 |  |  |  |  |
| 21 | 3 | 7.44 |  |  |  |  |
| 22 | 3.1 | 7.67 |  |  |  |  |
| 23 | 3.5 | 6.43 |  |  |  |  |
| 24 | 3.6 | 7.16 |  |  |  |  |
| 25 | 1.9 | 6.16 |  |  |  |  |
| 26 | 1.8 | 4.66 |  |  |  |  |
| 27 | 3.23 | 4.78 |  |  |  |  |
| 28 | 3.46 | 7.12 |  |  |  |  |
| 29 | 3.58 | 6.99 |  |  |  |  |
| 30 | 1.88 | 6.87 |  |  |  |  |
| 31 | 1.96 | 8.42 |  |  |  |  |
| 32 | 4.1 | 8.54 |  |  |  |  |
| 33 | 2.99 | 7.36 |  |  |  |  |
| 34 | 2.89 | 7.76 |  |  |  |  |
| 35 | 5.01 | 6.21 |  |  |  |  |
| 36 | 4.76 | 6.45 |  |  |  |  |
| 37 | 3.79 | 7.72 |  |  |  |  |
| 38 | 4.23 | 8.26 |  |  |  |  |
